# Impact of a workplace ‘sit less, move more’ program on efficiency-related outcomes of office employees

**DOI:** 10.1186/s12889-017-4367-8

**Published:** 2017-05-16

**Authors:** Anna Puig-Ribera, Judit Bort-Roig, Maria Giné-Garriga, Angel M. González-Suárez, Iván Martínez-Lemos, Jesús Fortuño, Joan C. Martori, Laura Muñoz-Ortiz, Raimon Milà, Nicholas D. Gilson, Jim McKenna

**Affiliations:** 1 Departament de Ciències de l’Activitat Física, Centre d’Estudis Socials i Socio Sanitaris, Universitat de Vic-Universitat Central de Catalunya, c/ Sagrada Família 7, 08500 Vic (Barcelona), Spain; 2 Physical Activity and Sport Sciences Department, FPCEE Blanquerna, Universitat Ramon Llull, c/Císter 34, 08022 Barcelona, Spain; 3 Physical Therapy Department, FCS Blanquerna, Universitat Ramon Llull, c/Padilla 326-332, 08025 Barcelona, Spain; 4 Departamento de Educación Física y Deportiva, Universidad del País Vasco, Portal de Lasarte 71, 01007 Vitoria, Spain; 5 Facultad CC.EE. e do Deporte, Universidad de Vigo, Campus A Xunqueira s/n, 36005 Pontevedra, Spain; 6 Department of Economics and Business, Universitat de Vic-Universitat Central de Catalunya, c/Sagrada Família 7, 08500 Vic (Barcelona), Spain; 7 Agència de Qualitat i Avaluació Sanitàries de Catalunya (AQuAS), c/Roc Boronat 81-95 (edifici Salvany), 2a planta, 08005 Barcelona, Spain; 8 Departament de Salut i AccióSocial, Universitat de Vic-Universitat Central de Catalunya, Vic (Barcelona), Spain; 9 School of Human Movement and Nutrition Sciences, The University of Queensland, Brisbane, St. Lucia Campus, 4072 Australia; 10 Carnegie School of Sport, Leeds Beckett University, Fairfax Hall, Headingley Campus, Leeds, LS6 3QS UK

**Keywords:** Presenteeism, Well-being, Workplace, Sitting time, Physical activity

## Abstract

**Background:**

Few studies have examined the impact of ‘sit less, move more’ interventions on workplace performance. This study assessed the short and mid-term impacts of and patterns of change within, a 19-week workplace web-based intervention (Walk@WorkSpain; W@WS; 2010–11) on employees´ presenteeism, mental well-being and lost work performance.

**Methods:**

A site randomised control trial recruited employees at six Spanish university campuses (*n* = 264; 42 ± 10 years; 171 female), assigned by worksite and campus to an Intervention (IG; used W@WS; *n* = 129; 87 female) or an active Comparison group (A-CG; pedometer, paper diary and self-reported sitting time; *n* = 135; 84 female). A linear mixed model assessed changes between the baseline, ramping (8 weeks), maintenance (11 weeks) and follow-up (two months) phases for the IG versus A-CG on (i) % of lost work productivity (Work Limitations Questionnaire; WLQ); (ii) three scales for presenteeism (WLQ) assessing difficulty meeting scheduling demands (Time), performing cognitive and inter-personal tasks (Mental-Interpersonal) and decrements in meeting the quantity, quality and timeliness of completed work (Output); and (iii) mental well-being (Warwick-Edinburgh Mental Well-being Scale). T-tests assessed differences between groups for changes on the main outcomes. In the IG, a multivariate logistic regression model identified patterns of response according to baseline socio-demographic variables, physical activity and sitting time.

**Results:**

There was a significant 2 (group) × 2 (program time points) interaction for the Time (F [3]=8.69, *p* = 0.005), Mental-Interpersonal (F [3]=10.01, *p* = 0.0185), Output scales for presenteeism (F [3]=8.56, *p* = 0.0357), and for % of lost work performance (F [3]=10.31, *p* = 0.0161). Presenteeism and lost performance rose significantly in both groups across all study time points; after baseline performance was consistently better in the IG than in the A-CG. Better performance was linked to employees being more active (Time, *p* = 0.041) and younger (Mental-interpersonal, *p* = 0.057; Output, *p* = 0.017). Higher total sitting time during nonworking days (Mental-interpersonal, *p* = 0.019) and lower sitting time during workdays (WLQ Index, *p* = 0.013) also improved performance.

**Conclusion:**

Versus an active comparison condition, a ‘sit less, move more` workplace intervention effectively reduced an array of markers of lost workday productivity.

**Trial registration:**

NCT02960750; Date of registration: 07/11/2016.

## Background

Eighty percent of adults in developed countries spend one third of their working day doing sedentary, desk-based tasks [1, 2]; this represents high exposure to the established health risk of uninterruptedly sitting for too long. Combining prolonged sitting with insufficient physical activity (PA) is strongly associated with all-cause mortality (hazard ratio: 4.23) [3]. However, replacing as little as 10 min of sedentary time with the same amount of light or moderate PA is associated to a lower prevalence of the metabolic syndrome (OR = 0.96 and OR = 0.89 respectively) [4], triglycerides (−1.9% for light PA; −8.5% for moderate PA) or insulin levels (−2.4% for light PA; −10.7% for moderate PA) [5]. In this context, an expert statement to promote better health among office employees has recommended to accumulate 2 h/day of standing and light walking during working hours, to eventually progress to a total accumulation of 4 h/day [6].

Several strategies have been used to reduce workplace sedentariness including (i) the use of sit-stand and treadmill work stations and, (ii) introducing incidental movement and short walks into the work routine [7–11]. Studies using active work stations have reported decreases in occupational sitting time [12, 13], increases in standing time [12] and occupational energy expenditure [14], improvements in cardiometabolic risk parameters (postprandial glucose and HDL cholesterol) [12, 15]; and reductions in fatigue levels and lower back discomfort [16]. Similarly, introducing incidental movement and short walks into the work routine have been reported to reduce occupational sitting, increase step counts [17–19] and reduce waist circumference [17]. However, standing and treadmill desk-based or ‘sit less and move more’ workplace interventions have showed mixed results for improving psychological well-being with little or no impact on work productivity [15–20]. Most research has investigated whether using active workstations in the office maintains employee performance, rather than enhancing employee productivity [8, 12, 14, 16].

Health-related productivity loss or presenteeism (time of impaired performance while at work due to health reasons) [21], was reported in 50% to 70% of European employees during 2010 [22]; leading to a decrement in the ability to function at work (−17.8% to −37.4%) and being potentially more costly than absenteeism and medical costs [23–25]. Higher presenteeism has been associated with (i) high sitting times before/after work and during lunch hours [26], and (ii) high occupational and total sitting time on workdays in highly active office employees [27]; suggesting that workplace strategies to improve the productivity of office employees should focus on reducing sitting time alongside efforts to increase PA [27]. Given the need to target specific employee behaviors that improve both health and efficiency-related outcomes; understanding whether workplace interventions for reducing occupational sitting promote productivity is a key issue.

Walk@WorkSpain (W@WS) is an automated evidence-based ‘sit less, move more’ office web-based intervention that successfully encouraged Spanish office workers to displace occupational sitting (−21 min/day sitting at work) with incidental movement and short walks (+1400 steps/day) [17]; eliciting sustained behavioral changes at two months follow up [17]. The present study is a secondary outcome of the original study [17], which builds on these findings and addresses limitations in the current evidence base. Specifically, we aim to evaluate the effectiveness of the W@WS program in relation to psychosocial outcomes for presenteeism and mental well-being in Spanish sedentary office employees.

## Methods

### Study design and sample

Methods, study population and recruitment procedures of the W@WS program have been previously detailed [17]. Briefly, the study used a site randomised control trial design. Participants were administrative and academic staff with low and moderate PA levels (0 to 3000 MET·min·wk.^−1^;International Physical Activity Questionnaire short form, IPAQ) [28] working at six campuses in four Spanish Universities: University of Vic-Central University of Catalonia (*n* = 1 campus) and University Ramon Llull-Blanquerna (*n* = 1 campus) for the Catalonia region, University of Vigo (*n* = 2 campuses) for the Galicia region and, University of the Basque Country (*n* = 2 campus) for the Basque Country region. Around 2500 emails were sent to target campuses. Office workers were first invited to participate in an on-line survey to identify those most in need of intervention (i.e., employees located at the low end of the PA volume continuum). A total of 704 employees completed the survey [27]. Employees with low or moderate PA levels (0 to 3000 MET·min·wk.^−1^) [28] were invited to participate in the intervention by email or phone calls (*n* = 345, 62%). Highly active employees (>3000 MET·min·wk.^−1^) [28] were excluded as they tend to spend less time sitting at work than their low or moderately active counterparts [1].

By region, an independent researcher generated a computer-based random sequence of targeted campuses, guaranteeing that one campus in each region was randomly assigned to the Intervention group (IG; deployed W@WS) or to the active Comparison group (A-CG; pedometer, paper diary and self-reported sitting time). A final sample of 264 academic and administrative staff were allocated to the A-CG (*n* = 3 campuses; *n* = 135 employees) and the IG (*n* = 3 campuses; *n* = 129 employees). Accepting an alpha risk of 0.05 in a two-sided test with 135 subjects for the ACG and 129 for the IG, a statistical power of 74% indicated that the sample size would detect a statistically significant difference in means of 2 units (4.2 in the ACG and 2.2 in the IG) for the percentage of Work productivity loss (WLQ Index Score).

Both groups were given a pedometer and a paper diary to register daily step counts and self-reported sitting time throughout the intervention. During delivery, the IG had access to the W@WS website program while the A-CG was asked to maintain habitual behaviour from September 2010 to June 2011, to fit within the academic year. Participants were blinded to the existence of either group. Contamination across groups was minimised by locating campuses in different cities and regions across Spain. Ethical approval was secured at each university by their ethics committees: Ethics Committee of the Faculty in Psychology, Education and Sport Sciences (University Ramon Llull); Research Commission of University of Vic; Ethics Committee of Clinical Research in Conselleria de Sanidad (CEIC; Xunta de Galicia); Ethics Committee of Applied Research in Human Beings (CEISH/GIEB; University of the Basque Country). Prior to the intervention, all recruits provided written informed consent.

### Intervention

W@WS is based on a generic automated web-based program which aims to encourage office employees to progressively ‘sit less and move more’ during workdays [17, 18]. The Spanish version of the intervention consists of a ramping phase (8 weeks) followed by a maintenance phase (11 weeks); with an overall program time duration of 19 weeks. During the ramping phase, tips were provided every two weeks to challenge employees to progressively increase their movement by 1000 to 3000 daily steps above baseline. In the first two weeks, breaking prolonged occupational sitting time through incidental movement during work tasks is the target. Subsequent weeks build on this ‘small changes’ approach by reducing overall sitting time through short walks (5–10 min), during morning/afternoon work breaks and/or commuting time (weeks 3–4); and longer walks (10 min or more) at lunchtime (weeks 5–6)*.* During weeks 7–8, employees are challenged to regularly achieve at least 10,000 daily steps, and also to increase walking intensity. During the maintenance phase (weeks 9–19), W@WS provides automated guidance with periodic emails encouraging sustained changes in sitting and walking, achieved in previous phases. The specific strategies used at different intervention stages are detailed elsewhere [10].

The W@WS website also provides a range of ecological support strategies to facilitate sitting time reductions and step count increases at work. These include (a) logging daily step counts into a personal account and receiving feedback on the achievement of goals through visual graphics and prompts, (b) social networking for sharing experiences and, (c) educational materials on the health benefits of ‘sitting less and moving more’ [10, 17].

An outcome evaluation of W@WS reported that IG participants decreased occupational sitting by 21 min/day while also increased step counts by 1400 steps/day compared to A-CG employees. This indicates that W@WS can be an effective, low-cost translational program to help Spanish sedentary, desk-based employees “sit less and move more” at work [17]. Most importantly, even at two months after withdrawing the IG continued averaging 16.5 min less sitting per day at work when compared to the A-CG [17]. A process evaluation of W@WS indicated that replacing sedentary occupational tasks with active work tasks through incidental movement and short walks had the potential to increase office employees´ everyday occupational PA without involving changes in the office environment [10]. Active work breaks, active travel and recording higher step counts were the approaches most frequently used for decreasing occupational sitting and increasing workplace walking [10].

### Data collection and measurements

Prior to intervention, a survey assessed participants´ physical activity levels (IPAQ short form; MET-minutes/week) [28], weekly total sitting time while traveling and, watching TV during working and nonworking days (minutes/day) [29], socio-demographic variables (age, gender, education, occupation [academic or administrative staff], working day [full time, part time or associated] employment contract [temporary, indefinite, civil servant, other]), presenteeism (Work Limitations Questionnaire; WLQ) [29], percentage of work productivity loss (WLQ Index Score) [30] and mental well-being (Warwick-Edinburgh Mental Well-Being Scale; WEMWBS) [31]. At each campus, trained and experienced researchers across sites provided the questionnaire to the IG and A-CG (i) during the first scheduled meeting (baseline; week 0), (ii) after the ramping phase (week 8), (iii) after the maintenance phase (week 19) and, (iv) at two months follow-up. Each participant was provided with standard detailed written information and instructions on completing the questionnaire. The researchers collected completed questionnaires at the end of the scheduled meeting for each phase and forwarded SPSS files electronically to a coordinating researcher who pooled and treated the data.

The *Work Limitations Questionnaire* (WLQ) assessed productivity and the degree to which health problems interfered with the ability to perform job roles [30]. The WLQ has been translated, adapted and validated for the Spanish and Catalan population [32]. In the WLQ, respondents self-report levels of difficulty in performing 25 specific job roles on a five point ordinal response scale ranging from “difficult all the time” to “difficult none of the time” across four scales; scores are expressed as an average of responses [30]. The 5-item (items 1–5) “Time Scale” addresses difficulty in scheduling demands. For the “Mental-Interpersonal Scale” nine items (items 6–14) cover difficulty performing cognitive tasks involving the processing of sensory information and a person’s problems interacting with others on-the-job; and the “Output Scale” has five items (items 15–19) exploring limitations in meeting demands for quantity, quality and timeliness of completed work. The six-item (items 20–25) “Physical Scale” assesses ability to perform job tasks that involve bodily strength, movement, endurance, coordination and flexibility [30].

Sub-scales scores are transformed to a 0–100 continuum to represent the percentage of time in the previous two weeks affected by limited on-the-job performance (from low to high rate of difficulty). At baseline 20 cases were deleted where ≥12 of the 25 item responses were missing. Where fewer data points were missing, intention-to-treat was applied and data imputed sequentially using the previously entered average from either baseline or the ramping (*n* = 49), maintenance (*n* = 83) and follow-up (*n* = 86) phases as appropriate. For isolated missing values, the average of sub-scale responses replaced the missing value. These sub scales scores can also estimate percentage of work productivity loss by obtaining an index score, known as the WLQ index; this is the weighted sum of the scores from the WLQ scales [30]. This calibrates the productivity impact of health-related work limitations based on the WLQ index score [30]. Thus, a WLQ index score of −5 represents a 4.9% decrease in productivity; 5.1% additional work hours are needed to compensate for this level of productivity loss [30]. In the present study, the WLQ index was calculated by summing the scores of three WLQ scales; the “Physical Scale” was excluded from the current analyses as it was irrelevant to these job roles.

The Warwick-Edinburg Mental Wellbeing Scale (WEMWBS) assessed positive mental well-being (positive functioning, happiness and subjective wellbeing) over the previous two weeks [31]. This 14-item scale has five response categories; 1 (“None”) to 5 (“All the time”). Responses are summed to identify the final score, 14–70, indicating low to high positive mental well-being. WEMWBS shows high internal reliability (Cronbach’s alpha = 0.93) and one-week test-retest reliability (*r* = 0.97) in the Spanish population [33]. For WEMWBS, missing values were detected from baseline to ramping (*n* = 10, *n* = 16 for the ACG and IG respectively), to maintenance (*n* = 18; *n* = 21 for the ACG and IG respectively) and follow-up (*n* = 5; *n* = 3 for the ACG and IG respectively). For isolated missing values, the average of sub-scale responses replaced the missing value.

### Statistical analyses

A descriptive analysis of the subjects’ characteristics was performed using proportions and measures of central tendency and dispersion according to the nature of the variables. Differences between groups for changes in the main outcomes across program time points were assessed using Student’s t-tests.

A linear mixed model assessed changes within groups in the scores of the three scales for presenteeism (Time, Mental-Interpersonal, Output), the percentage of work productivity loss (WLQ index) and the scores for mental well-being (WEMWBS scale) across the four program time points (baseline, ramping, maintenance and follow-up). The model was adjusted by gender and age. The design of the model included participants (fixed factor), group (experimental and comparison group) and program time points (baseline, ramping, maintenance and follow-up). When the interaction between program time points*group was significant, changes 2 × 2 were assessed using post-hoc test adjusted by the Sidak method. In the IG, a multivariate logistic regression model identified patterns of response to the intervention by assessing differences on participants´ socio-demographic, physical activity and sitting time levels at baseline. The model was adjusted by baseline scores in the Time, Mental-Interpersonal, Output scale, WLQ index and mental well-being respectively. Preliminary checks ensured no violation of assumptions of normality, homogeneity of variance and homogeneity of regression slopes. These analyses were performed using a PROC MIXED procedure in SAS version 9.3 (SAS Institute Inc., Cary, NC, USA).

## Results

### Pre-intervention characteristics

A total of 264 employees were recruited (42 ± years of age; *n* = 171 women; *n* = 129 administrative staff). In Catalonia, 115 people agreed to participate (IG = 63), with 109 in the Basque Country (IG = 44) and 40 in Galicia (IG = 22). Table 1 shows participants´ socio-demographic variables, physical activity and sitting time according to the IG and A-CG.

**Table 1:** Main characteristics of participants in the Walk@WorkSpain study by socio-demographic variables according to the active Comparison and Intervention groups

	Comparison n = 135	Intervention n = 129
Sex, n (%)		
Men	51 (37.8)	42 (32.6)
Women	84 (62.2)	87 (67.4)
Age, Mean (SD)	43 (11)	41 (9)
Education, n (%)		
Do not have regulated studies	1 (0.8)	1 (0.8)
Secondary mandatory school or equivalent	1 (0.8)	1 (0.8)
High school	9 (6.9)	3 (2.3)
Apprentice	4 (3.0)	1 (0.8)
Professional training	10 (7.6)	7 (5.5)
University degree or superior	106 (80.9)	115 (89.8)
University, n (%)		
University of Vic-Central University of Catalonia	0 (0.0)	63 (48.8)
University of Vigo	18 (13.3)	22 (17.1)
University Ramon Llull – Blanquerna	52 (38.5)	0 (0.0)
University of the Basque Country	65 (48.2)	44 (34.1)
Occupation, n (%)		
Academic	60 (45.8)	71 (55.5)
Administrative	71 (54.2)	57 (44.5)
Working day, n (%)		
Full time	104 (79.4)	110 (85.9)
Part time	26 (19.8)	16 (12.5)
Associated	1 (0.8)	2 (1.6)
Employment contract, n (%)		
Temporary	34 (26.0)	25 (19.5)
Indefinite	49 (37.4)	56 (43.8)
Civil servant	44 (33.6)	42 (32.8)
Other^a^	4 (3.0)	5 (3.9)
Physical Activity (MET-minutes/week), mean (SD)	3445.04 (2778.85)	2648.84 (2201.17)
Sitting time traveling (minutes/day)		
Weekday	89.23 (54.91)	76.12 (50.14)
Weekend day	77.57 (50.46)	77.46 (53.82)
Sitting time watching TV (minutes/day)		
Weekday	106.78 (61.77)	85.69 (71.27)
Weekend day	179.39 (101.61)	145.48 (92.55)
Total sitting time (minutes/day)		
Weekday	518.71 (138.64)	516.62 (156.72)
Weekend day	326.19 (171.39)	326.22 (167.01)

*SD* Standard Deviation

^a^Scholar contract

A flowchart of participant recruitment across all phases of the W@WS intervention has been described in detail elsewhere [17]. Briefly, 244 (92%) employees at baseline completed full data measurements for the scales of presenteeism, percentage of work productivity loss and mental well-being. Full data sets from baseline through the ramping period were provided by 215 (81%) participants, while 181 (68%) provided full data sets through the maintenance period. One hundred and seventy-eight participants (67%) completed 19 weeks of data from baseline through follow-up.

### Intervention effects on the scales for presenteeism, percentage of work productivity loss and mental well-being

There was a significant 2 (group) × 2 (program time points) interaction for the Time (F [3]=8.69, *p*-value interaction = 0.005), Mental-Interpersonal (F [3]=10.01, *p*-value interaction = 0.0185), and Output scales for presenteeism (F [3]=8.56, *p*-value interaction = 0.0357), and for the percentage of work productivity loss (F [3]=10.31, *p*-value interaction = 0.0161; Table 2). In both the IG and the A-CG, presenteeism and losses in work productivity were significantly greater across the program, indicating a universal increase in the difficulty of achieving scheduling demands (Time scale; Table 2), performing cognitive tasks as well as interacting with others (Mental-interpersonal scale; Table 2) and in meeting demands for quantity and quality of completed work (Output scale; Table 2). However, levels of performance impairment were smallest in the IG, versus the A-CG, across program time points.

**Table 2:** Outcome measures (Scales for Presenteeism, Percentage of Work Productivity Loss and Mental Well-Being during the program phases within the Intervention (used W@WS) and active Comparison group (used a pedometer, paper diary and self-reported sitting time)

	Baseline		Rampling phase^a^		Maintenance phase^b^		Follow-up^c^		*P* values		
	Comparison (*n* = 135)	Intervention (*n* = 129)	Comparison (*n* = 125)	Intervention (*n* = 112)	Comparison (*n* = 107)	Intervention (*n* = 91)	Comparison (*n* = 102)	Intervention (*n* = 88)	Group	Program time points	Interaction
Presenteeism (WLQ)^d^											
Time scale^e^											
Mean	56.02	68.97	71.54	79.94	75.69	79.09	78.89	80.90			
95% CI	52.0 to 61.0	64.8 to 73.1	67.5 to 75.6	75.9 to 84.1	71.6 to 79.7	74.9 to 83.2	74.8 to 82.9	76.7 to 85.0	0.005	<0.001	0.005
Mental-interpersonal scale^f^											
Mean	54.22	65.01	70.42	76.29	74.72	76.81	75.48	76.75			
95% CI	50.5 to 58.0	61.2 to 68.8	66.7 to 74.1	72.5 to 80.1	71.0 to 78.4	73.0 to 80.6	71.8 to 79.2	72.9 to 80.5	0.034	<0.001	0.018
Output Scale^g^											
Mean	42.96	50.14	54.10	56.80	57.19	58.35	59.58	58.79			
95% CI	40.0 to 46.0	47.0 to 53.2	51.1 to 57.1	53.7 to 59.8	54.1 to 60.2	55.3 to 61.4	56.5 to 62.0	55.7 to 61.8	0.211	<0.001	0.035
Percentage of lost work productivity WLQ Index Score^h^											
% of Work productivity loss (WLQ Index Score)											
Mean	12.61	14.95	17.51	17.91	18.00	17.85	17.94	17.69			
95% CI	11.8 to 13.4	14.2 to 15.7	16.6 to 18.3	17.1 to 18.7	17.1 to 18.9	16.9 to 18.7	17.0 to 18.8	16.8 to 18.6	0.185	<0.001	0.016
Mental Well-Being at Work (WEMWBS)^i^											
Mental Well-Being											
Mean	61.03	59.83	56.48	55.96	55.76	56.06	55.68	55.44			
95% CI	59.9 to 62.1	58.7 to 60.9	55.3 to 57.6	54.8 to 57.1	54.6 to 56.8	54.9 to 57.2	54.5 to 56.6	54.2 to 56.6	0.716	<0.001	0.305

^a^After the ramping phase week 8, ^b^After the maintenance phase week 19, ^c^At two months follow-up

^d^Each scale score indicates the percentage of time in the previous two weeks when the respondent was limited in performing a specific dimension of job tasks (from low to high rate of difficulty in performing job demands). The minimum score is 0 (limited none of the time) to 100 (limited all of the time)

^e^Five items addressing difficulty in scheduling demands

^f^Six items covering difficulty in performing cognitive tasks involving the processing of sensory information and a person’s problems interacting with people on-the-job.

^g^Five items addressing decrements in the ability to meet demands for quantity, quality and timeless of completed work

^h^A percentage estimate of work loss based on the weighted sum of the scores from the Work Limitations Questionnaire WLQ scales

^i^Warwick-Edinburgh Mental Well-being Scale WEMWBS: Scores range 14 to 70. Higher scores indicate better positive mental well-being

Without intervention, the average percentage of time feeling limited in performing job tasks increased in the Time, Mental-Interpersonal and Output scales scores from baseline through ramping (+15.5; +16.2; +11.1 respectively), maintenance (+19.7; +20.5; +14.2 respectively) and follow-up (+22.9; +21.3; +16.6 respectively). In the IG, equivalent patterns, but at a smaller scale, were found from baseline through ramping (+11; +11.3; +6.7), maintenance (+10.2; +11.8; +8.2) and follow-up (+12; +11.7; +8.7).

**Fig. 1:**
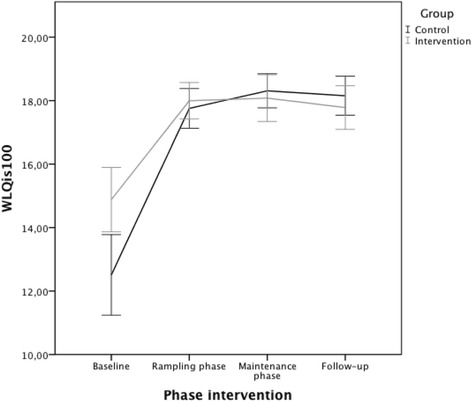
Change in the average percentage of work productivity loss for the intervention and comparison groups across program phases (WLQ Index Score)^1^. ^1^An increase in the percentage of lost work productivity (WLQ Index Score) means a decline in job productivity

Similarly, the A-CG reported a larger impairment in the percentage of lost work productivity (WLQ Index Scores) from baseline through ramping (+4.9), maintenance (+5.4) and follow-up (+5.3; Fig. 1) when compared to the IG; baseline to ramping (+3), maintenance (+2.9) and follow-up (+2.8) (Fig. 1). Significant mean differences between groups were found for changes from baseline to maintenance in Time (+9.5; *p* = 0.017), Mental-interpersonal (+8.5; *p* = 0.019), the Output scale scores (+6; *p* = 0.041) and the WLQ index scores (+1.7; *p* = 0.021). Mean differences between groups on the main outcomes remained significant at two months follow-up (Time + 10.9, *p* = 0.007; Mental-interpersonal +9.6, *p* = 0.009; Output +7.9, *p* = 0.006; WLQ index +2, *p* = 0.005); with the A-CG consistently showing higher presenteeism impairments and job productivity loss compared to the IG across time points.

**Fig. 2:**
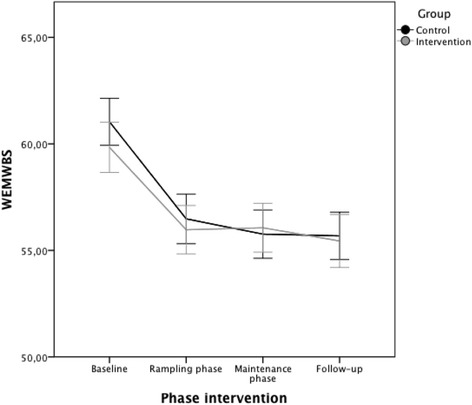
Change in mental well-being (mean) for the intervention and comparison groups across program phases (WEMWBS)^1^. ^1^Warwick-Endinburgh Mental Well-being Scale (WEMWBS). The minimum score is 14 and the maximum is 70. A decrease in WEMWBS scores means a decline in mental well-being

Differences in the WLQ index scores between groups at maintenance and follow-up (+1.7; +2 respectively) favoured the IG in both phases (+1.67% and 2.04% respectively) over the A-CG [29]. No significant interactions were identified between group and program time points for mental well-being (Fig. 2). Between the private and publicly funded university sites, there were no differences in any outcome measures.

### Patterns of response to the intervention

Among the 129 IG participants, 45.7% (*n* = 59) reported beneficial levels of performance in the Time, Mental-Interpersonal (53.5%, *n* = 69) Output scales (55%, *n* = 71) and WLQ index scores (38%; *n* = 49) while 27.1% (*n* = 35) reported beneficial levels in mental well-being. Overall, 58.1% (*n* = 75) of W@WS participants reported benefits in at least one marker of work efficiency.

Looking at differences in baseline versus follow up presenteeism, beneficial levels of performance were identified in the more active employees (time scale, +643 MET-minutes/week; 2353 ± 2016 vs. 2999 ± 2372 METs-minutes/week; *p* = 0.041); with higher total sitting time during nonworking days (mental-interpersonal scale, +70 min/day; 290 ± 158 vs. 360 ± 168 min/day; *p* = 0.019), attributable to more minutes of weekend watching TV (120 ± 72 vs. 169 ± 100 min/day; *p* = 0.064); in the younger employees (mental-interpersonal scale, −3 years of age, 43 ± 9 vs. 40 ± 9 years of age, *p* = 0.057; output scale, −3 years of age 43 ± 10 vs. 40 ± 8 years of age, *p* = 0.017 respectively) and, with lower sitting time during working days (WLQ Index, −88 min/day; 549 ± 158 vs. 461 ± 136 min/day; *p* = 0.013).

## Discussion

This study assessed the impact of a ‘sit less, move more’ workplace intervention on presenteeism, health-related productivity loss and mental well-being over 19 weeks and at two months follow-up. Three main findings were identified on the impact of W@WS on efficiency-related outcomes. First, an automated internet-delivered intervention attenuated presenteeism in employees who engaged W@WS, sustaining these effects two months after program completion. Second, with a similar pattern over time, the percentage of losses in health-related productivity in employees engaging W@WS was less than in the comparison group at follow up. Third, engaging in W@WS had no distinctive effect on employees´ mental well-being. A tripartite rational justifies targeting behavioural risk factors associated with high presenteeism; (i) chronic diseases affect a high proportion of the adult population worldwide [34], (ii) productivity losses from employee presenteeism exceed job wages [24] and, (iii) up to 60% of all costs for 10 health conditions including hypertension ($392 per eligible employee per year), heart disease ($368), depression ($348), and arthritis ($327) [25].

The main result of the current study indicated that W@WS represents an effective low-cost translational program on attenuating some efficiency-related outcomes in desk-based employees. This is important because reducing performance was endemic across the sites and time course of the study*.* Without W@WS to compensate for these differences, and this was shown in the A-CG, employees would need to increase their work hours by 1.7% and 2.04% respectively [29]. This provides clear evidence of the direction and scale of the intervention effect for reducing losses in performance that were endemic to Higher Education. Such losses might be explained because post-intervention and follow-up measurements were taken during April and June. This is the busiest time of the year in Spanish universities as it’s the end period of degree courses with lots of exams, marking assignments, tutorials, enrolment of the new students for next academic year among others.

Employees using W@WS, which focuses on a simple ‘sit less, move more’ message spent less time feeling limited in (i) performing their job time and scheduling demands, (ii) cognitive and interpersonal tasks, and (iii) meeting demands for quantity, quality and timeliness of completed work than their counterparts. Most importantly, differences in the percentage of time feeling limited in performing job tasks were even greater at two months follow up. As a result, employees who engaged W@WS showed consistently smaller losses in percentage of work productivity loss across program time points than employees in the comparison group. Uniquely, this study builds on previous cross-sectional results [27] by contributing evidence showing that efficiency-related outcomes also differ depending on employees´ physical activity level, time spent sitting during nonworking days and total time spent sitting during working days, including outside working hours. Future research should investigate the impact of strategies to reduce workplace sitting time on work performance and mental well-being among employees engaged in different levels of pre-existing PA as well as time spent sitting both at weekends and on weekdays but outside working hours.

To our knowledge, only one equivalent study has examined a similar intervention, identifying no impact at all [13]. In that study, a randomized cross-over trial was based on a small sample (*n* = 28) of sedentary office workers who used sit-stand desks across 4-weeks of intervention [13]. Presenteeism was measured as a secondary outcome by the Work Productivity and Activity Impairment Questionnaire and did not provide any follow up measurements. W@WS addresses limitations in existing evidence by: (i) measuring presenteeeism with the best instrument reported (Work Limitations Questionnaire) [35], (ii) comparing the results against a comparison group, the best scientific design for identifying which interventions achieve the best effects and [36], (iii) providing evidence-based data at follow-up from a translational program that can be applied in every day sedentary occupations.

Most evidence on the effectiveness of reducing occupational sitting on employees´ job productivity has addressed how much using sit-stand or treadmill work stations *negatively* influenced work-related productivity; focusing on its overall acceptability within the workplace [12]. Several studies have identified that cognitive performance is not impaired by short-term use of these workstations (working while standing, or while walking at low intensity) [8, 16, 37, 38], indicating that using these devices do not compromise employees´ work-related productivity if there is a good alignment with job tasks [12–14]. While Tudor-Locke et al. (2014) [7] indicated that little was known about the impact of learning or adaptation on using active workstations on employees´ work productivity, W@WS now offers preliminary evidence of sustained positive work-oriented outcomes linked to using “sit less, move more” programs during working hours.

The second main result of the current study indicated that engaging in W@WS demonstrated no distinctive effect on improving employees´ mental well-being; existing cross-sectional data indicates a range of diverse associations. Decreased mental well-being was linked to higher sitting times during work days and occupational sitting in highly active Spanish office employees [27]. In New Zealand, optimal wellbeing was more common among adults reporting sitting “almost none of the time” (1.87, 1.01–3.29, *p* < 0.01) [39]. In UK women, total non-occupational sitting time was adversely associated with mental well-being [40], while in the English adult population, those classified in the highest tertile of objectively measured sedentary time had the highest risk of psychological distress (multivariate adjusted OR = 1.74, 95% CI 1.07 to 2.83) compared to those in the least sedentary tertile [41]. In addition, a recent systematic review on the effect of standing and treadmill desks interventions for improving psychological well-being has shown mixed results; studies (*n* = 7) mainly measured employees´ mood state rather than mental well-being [15].

Remarkably few studies have focused on the impact of ‘sit less, move more’ programs on employees´ mental well-being. In our study, participants showed low variability within high baseline mental well-being scores (61 points and 59.8 points in the WEMMBS score out of 70 for the A-CC and IG respectively). High baseline scores – probably inflated by employees having just returned from their summer holidays - may have created a possible ceiling effect, making further improvements difficult to achieve. Another possible reason for securing no increases in well-being could be that universal fluctuations affected the whole sector as the global economic recession hit Spain. Future research should study these impacts in samples with wider variations in mental well-being scores, including participants with lower-end scores.

Furthermore, it is possible that the scale of changes on occupational sitting (−21 min/day) and step counts (+1400 steps/day) linked to W@WS were insufficient to elicit measurable improvements on mental well-being. Future research should test the impact of workplace interventions for reducing occupational sitting on employees´ mental well-being using objective measurements of sitting time; self-reported measurements may lack sensitivity to detect all changes in occupational sitting. Nonetheless, participants who reported beneficial levels of mental well-being at follow up (*n* = 35) were those with higher baseline presenteeism on all three subscales (*p* < .05). This means that the intervention effect was strongest in those whose initial work performance was most impaired. Future research should also study these impacts in samples showing high presenteeism at baseline.

This study has several strengths and limitations. First, self-report estimates always have the potential for error, even though the measures used in the current study had high validity and reliability. Given that the WLQ is one of the three most suitable instruments to explore the links between PA and presenteeism in workplace PA research [34], our study contributes to the scarce evidence by exploring intervention effectiveness using a tool with acceptable measurement properties. Second, it is important to recognize that this test of W@WS was based on highly educated, low-to-moderately active (as highly active employees were excluded) and, middle-aged university employees. Ongoing research should focus on more heterogenous samples of employees from a range of workplaces with a wide range of mental well-being, presenteeism scores and PA levels. However, the attenuation seen in presenteeism and health-related productivity loss are encouraging; these may also be anticipated for desk-based occupations with equivalent administrative demands regardless of their overall organizational focus. In this regard, W@WS represents a contribution to implementation research that is needed to enhance population efficiency-related outcomes.

## Conclusions

W@WS represents a new evidence-based intervention that successfully mitigated office employees´ presenteeism and productivity work losses. These results were secured against an active comparison group, meaning that the work makes a unique contribution to addressing the short and mid-term impact of workplace interventions for reducing occupational sitting on promoting efficiency-related outcomes as well as identifying patterns of response to the intervention. Most importantly, W@WS elicited sustained changes on presenteeism over time, indicating both its feasibility and effectiveness for promoting work productivity in sedentary workplaces. Further, the strategies provided by, and the outcomes attributable to, W@WS were achieved without making major changes in the work environment.

## Abbreviations

A-CG: Active Comparion Group
IG: Intervention Group
IPAQ: International Physical Activity Questionnaire
METs: Metabolic Equivalent Units
PA: Physical Activity
TV: Television
UK: United Kingdom
W@WS: Walk@WorkSpain
WEMWBS: Warwick-Edinburg Mental Well-Being Scale
WLQ: Work Limitations Questionnaire
